# Determination of subpicogram levels of airborne polycyclic aromatic hydrocarbons for personal exposure monitoring assessment

**DOI:** 10.1007/s10661-023-10953-z

**Published:** 2023-02-07

**Authors:** Barend L. van Drooge, Raimon M. Prats, Clara Jaén, Joan O. Grimalt

**Affiliations:** Dept. Environmental Chemistry, Institute of Environmental Assessment and Water Research, Spanish National Research Council (IDÆA-CSIC), c/Jordi Girona 18-26, 08034 Barcelona, Spain

**Keywords:** Polycyclic aromatic hydrocarbons, Air pollution, High resolution mass spectrometry, GC-Orbitrap-MS, Aethalometer, Personal exposure monitoring

## Abstract

A method based on the use of GC coupled to Q-exactive Orbitrap mass spectrometry (GC-Orbitrap-MS) has been developed for the analysis of polycyclic aromatic hydrocarbons (PAHs) at sub-picogram levels. Outdoor ambient air particulate matter (PM2.5) and standard reference materials (SRM2260a) were analyzed in full scan mode showing low instrumental uncertainties (1–22%) and high linearity over a wide concentration range (0.5 pg and 500 pg/μL). Good reproducibility was obtained compared to the use of conventional single quadrupole GC–MS of PM samples. The quantification limit of the GC-Orbitrap-MS method for full scan analysis of PAHs in outdoor ambient air PM samples was 0.5 pg/μL. This low limit allowed the analysis of PAHs in samples collected with low volumes (< 0.5 m^3^), such as punch samples from whole filters or filter strips from personal exposure monitoring equipment. PAHs were successfully analyzed in filter strips from real-time Aethalometer AE51 equivalent black carbon (eBC) analyzers used in urban and rural sites, and in personal exposure monitors of firefighters during prescribed burns. The correlations between PAHs and eBC in these analyses were very strong (*r*^2^ ≥ 0.93). However, the equations obtained reflected the dominance of different emission sources, such as traffic in urban areas, wood burning for domestic heating, or wildfires. The method reported here affords the analyses of PAHs in high precision studies of atmospheric PM samples, e.g., high frequency sampling of low volumes, affording personal exposure monitoring assessments.

**Supplementary Information:**

The online version contains supplementary material available at 10.1007/s10661-023-10953-z.

## Introduction

Gas chromatography-mass spectrometry (GC–MS) is widely used for the detection and quantification of semi-volatile organic compounds in environmental samples, including atmospheric particulate matter (PM). It provides analytical advantages such as high chromatographic sensitivity, resolution, and reproducibility, as well as extensive spectral libraries to support the identification of chemical constituents. However, the understanding of the processes related with atmospheric pollution is progressively requiring analysis of smaller sample volumes for elucidation of specific sources and chemical reactions in real-case atmospheres, as well as personal exposure, which in turn demand for methods providing higher sensitivity and lower detection and quantification limits.

Over the past decade, the development and use of Orbitrap-based mass spectrometry for liquid chromatography has afforded good progress in compound identification and high-resolution analysis (Eliuk & Makarov, 2015). In 2015, GC coupled to Q-Exactive orbitrap mass spectrometry (GC-Orbitrap-MS) became commercially available. This instrument offers better sample characterization of organic compounds in GC amenable extracts while improving sensitivity in relation to other GC–MS approaches, including organic contaminants in environmental and food samples (Meng et al., 2022; Wickrama-Arachchige et al., 2020; Yang et al., 2019). In the present study, these properties are tested for the analysis of polycyclic aromatic hydrocarbons (PAHs) in sample extracts of atmospheric particulate matter (PM) collected on filters.

PAHs are ubiquitous environmental contaminants. They are components of fossil fuels, but their widespread environmental occurrence is related with incomplete combustion of organic materials (Howsam & Jones, 1998; van Drooge, 2013). Important sources of these aromatic hydrocarbons are, therefore, combustion of fossil and modern (biomass, e.g., wood) fuels for the generation of energy, but also wildfires and open organic waste combustion in agricultural fields. In many urban areas, exhausts from motorized vehicles with combustion engines are the most important emission source contributors, while domestic heating by wood combustion is a dominating emission source in many rural communities, essentially during the winter period and especially in forested areas, such as mountain valleys (Jaén et al., 2021; Puxbaum et al., 2007; van Drooge & Grimalt, 2015). Occasionally, wildfires are important emission sources for PAHs (Navarro et al., 2017), and wildfire smoke can dominate even urban air quality (van Drooge et al., 2016). Conventional sampling, detection, and quantification of these compounds in PM is performed by passing air volumes through a filter during several hours, subsequent extraction of these filters by organic solvents, clean-up, and PAH determination by gas or liquid column chromatography coupled to mass spectrometry or fluorescence detection systems, respectively (CEN/TS 16645:^2014^; EN 15549:^2008^; ISO 16362–2005; ISO 12884:^2000^). The sensitivity of quadrupole GC–MS and high-performance liquid chromatography (HPLC) techniques is suitable for high exposure PAH concentrations, but large sample volumes and sampling times are necessary to be able to detect the ambient air levels between few pg/m^3^ and several ng/m^3^. This often results in limits of quantification that are too high to be suitable for personal exposure monitoring of PAHs, especially the particle bounded high molecular compounds (Cherry et al., 2021; Maitre et al., 2018; Navarro et al., 2017; Nowakowski et al., 2022).

Analysis of PAHs at these relatively low concentrations is necessary since PAHs are mutagenic and carcinogenic, endocrine disruptors, have inflammatory properties (van Drooge, 2013) and are deleterious for neurodevelopment (Mortamais et al., 2017). The annual target level of ambient air PM for benzo[a]pyrene is set at 1 ng/m^3^ by the EU legislation (2004/107/EC), while the World Health Organization considers 0.12 ng/m^3^ as the reference level for the impact on human health (WHO, 2017). Accurate determination of trace PAH levels in atmospheric PM is therefore important for exposure studies, risk assessment, and regulation policies. The need for this information increases as evidence on the harmful health effects of air pollution increases (GBD, 2018). These requests also involve the use of smaller sample amounts to study the effects of secondary reaction on the PAH distributions.

In the present study, a method using GC-Orbitrap-MS has been developed for the determination of PAHs in atmospheric particles. Standard reference material (SRM) has been used for assessment of the analytical uncertainty of the PAH analyses. Real PM samples have also been used to evaluate reproducibility and repeatability. Finally, it has been demonstrated that the method is useful for the analysis of filter strips from Aethalometers (AETHLABS, San Francisco, USA) that collect samples of relatively small volumes (100 mL/min) for real-time equivalent black carbon (eBC) measurements in air quality and personal exposure monitoring assessments. Former studies with gas-chromatograph–atmospheric-pressure laser ionization–time of flight mass spectrometer (GC-APLI-TOF–MS) showed that these filter strips are suitable for PAHs analysis in indoor air (Stader et al., 2013; Yan et al., 2018). Here, we analyze the filter strips from Aethalometer AE51 that sampled outdoor air eBC in urban and rural sites, as well as the exposure of firefighters to smoke during a prescribed burn.

## Methodology and materials

### Reagents

The solvents cyclohexane, n-hexane, dichloromethane, methanol, and toluene were from Merck (KGaA, Darmstadt, Germany). The Standard Reference Material SRM2260a dissolved in toluene was from the National Institute of Standards and Technology (NIST; Gaithersburg, USA). Surrogate standards with sixteen deuterated PAH compounds dissolved in cyclohexane were from Dr. Ehrenstorfer (Augsburg, Germany).

### High volume PM samples

PM2.5 (atmospheric particulate matter with an aerodynamic diameter smaller than 2.5 µm) was collected on pre-cleaned quartz filters (Pall Corporation, New York, USA) in 2014 in the city of Manlleu (Catalonia, Spain) by means of a high-volume sampler CAV-A/MSB (MCV, Collbató, Catalonia, Spain) at a flux of 30 m^3^/h for 24 h. After sampling, the filters were stored at − 20 °C before analysis. This procedure is in line with the standardized methodology for PM sampling (EN 12341:1998; 1999/30/EC).

### Low volume PM samples

**Fig. 1:**
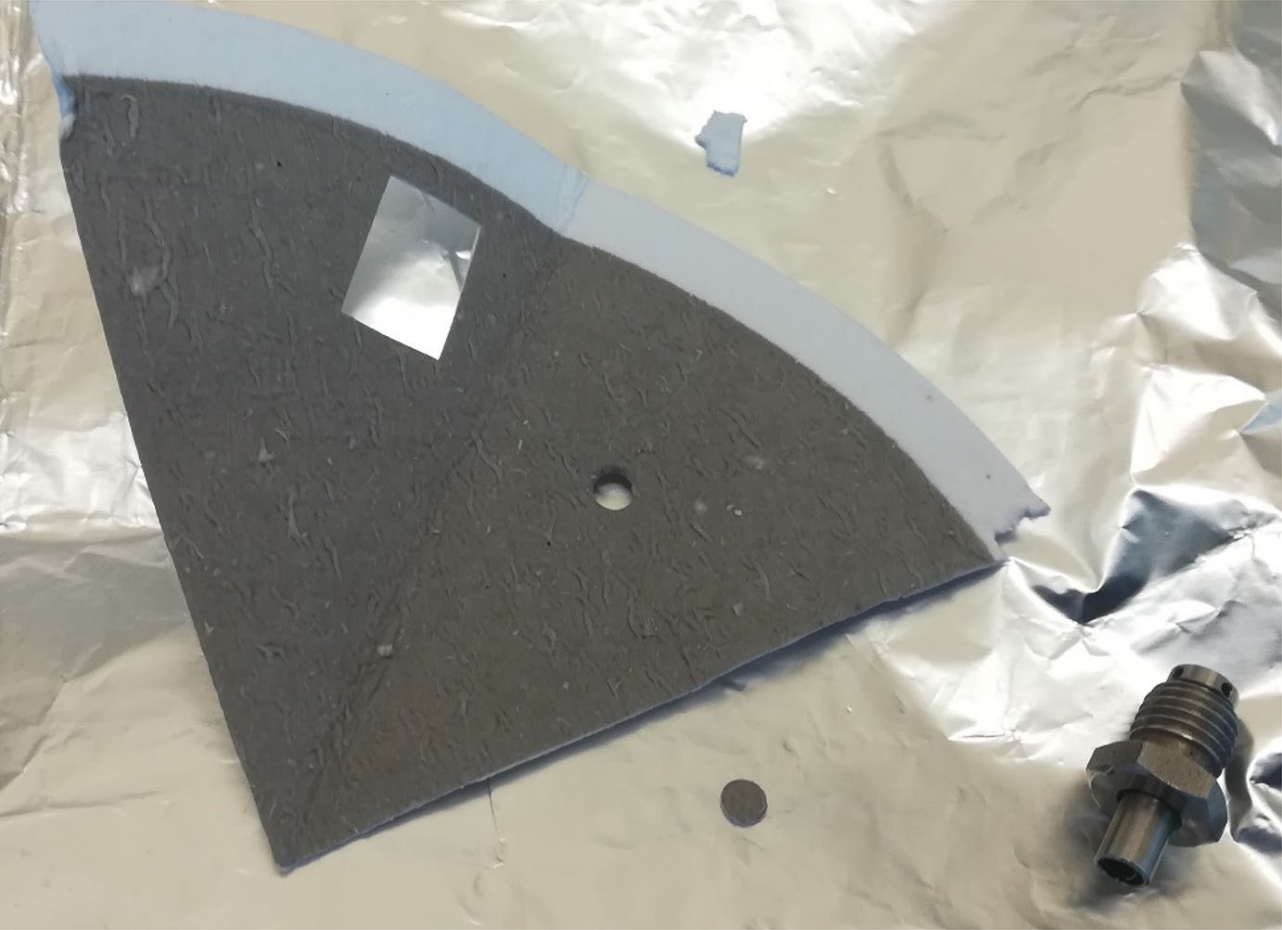
Image of 1/4 PM2.5 filter and sub-cubic meter fraction of sample taken by a punch cutter (Ø = 3.4 mm) that were analyzed by GC-Orbitrap-MS

A section of a high-volume PM quartz filter, equivalent to a sample volume of 0.37 m^3^, was taken by means of a punch cutter (Ø = 3.4 mm; Fig. 1) and was introduced into a 1.5-mL conical vial. The filters were spiked with 25 μL of surrogate PAH-D standard in cyclohexane (1 ng/μL) and were then extracted in an ultrasonic bath for 15 min. Filter fractions were removed from the vials before analysis by GC-Orbitrap-MS.

Other low volume PM2.5 samples were collected with an Aethalometer AE51 (AETHLABS, San Francisco, USA). This real-time, pocket-sized instrument is designed for personal exposure monitoring of equivalent black carbon (eBC). The instrument measures the rate of change in absorption of transmitted light (at 880 nm) during continuous sampling at 100 mL/min of aerosols (PM2.5) that are then deposited on a small filter strip. The collected PM is a 3 mm spot on the filter strip that is made of T60 Teflon-coated borosilicate glass fiber that is easily replaced. Samples were collected on these filter strips while they measured real-time eBC concentrations (at 5-min resolution for 24 h) in a rural site in the Pyrenees (La Pobla de Lillet, Catalonia, Spain; *n* = 11), an urban street site (Barcelona, Catalonia, Spain; *n* = 19) in wintertime, and personal exposure of firefighters during a prescribed forest burn (Avinyó, Catalonia, Spain; *n* = 2) (at 5-min resolution for 5 h). The sample spot was taken from the filter strip using a punch cutter (Ø = 3.4 mm), placed in a 1.5-mL vial (Fig. S1), and extracted in 25-μL surrogate standard solution containing deuterated PAHs in cyclohexane. Filter fractions were removed from the vials before analysis by GC-Orbitrap-MS.

### Analysis by conventional liquid extraction and GC–MS

The methodology applied for PAH analysis by solvent extraction, clean-up, and analysis by GC–MS is in accordance with standardized procedures (CEN/TS 16645:2014; EN 15549:2008; ISO 12884:2000). One quarter of the filters (180 m^3^) were analyzed in 2015 after being weighted on a microbalance for obtaining their exact volume equivalent. The portions of the filters were spiked with 25-μL surrogate PAH-D standard (1 ng/μL) and were then extracted in 15-mL solvent mixture of dichloromethane:methanol (1:1 v/v) in an ultrasonic bath for 15 min. After that, the extracts were passed to a flask through a syringe filter (Sartorius, Göttingen, Germany) to remove particles. This step was repeated twice. The final extract was concentrated to 0.5 mL by rotary evaporation. For the analysis of PAHs, the extract was introduced into an aluminum oxide (1 g) column whose adsorbent had been muffled the night before to 450 °C. The PAH fraction was obtained by elution with 4 mL of dichloromethane:hexane mixture (2:1 v/v), concentrated to 0.5 mL by rotary evaporation, and further concentrated to almost dryness under a gentle nitrogen gas stream.

Sample extracts were injected (1 μL) in a Trace GC Ultra–DSQ II single quadrupole MS (GC–MS) (Thermo Fischer Scientific, Whaltham, USA) equipped with a 30-m fused capillary column (HP–5MS, 0.25 mm × 25 μm film thickness). The oven temperature program started at 90 °C and held for 1 min, and then heated to 120 °C at 12 °C/min and to 320 °C at 4 °C/min, and held for 15 min. The injector, ion source, quadrupole, and transfer line temperatures were 280 °C, 200 °C, 150 °C, and 270 °C, respectively. Helium was used as carrier gas at 0.9 mL/s. A mass selective detector operating in single ion monitoring EI + (70 eV) mode was used for the analysis of individual PAHs. They were identified at the retention times of authentic SRM2260a standard on the GC column and by the ions that are given in Table 1. Quantification was performed by calculating the concentrations from the relationship between the chromatographic area of the ion peaks of the compounds to those of the ion peaks of the surrogate standards (area/area-D response) in the samples compared to amount/amount-D responses in a five-point external calibration curve.

**Table 1:** Studied PAHs and ion m/z analyzed by GCMS and GC-Orbitrap-MS

Compound	GCMS	GC-Orbitrap-MS
Biphenyl	154	154.0774
Naphthalene	128	128.0620
Acenaphthylene	152	152.0619
Acenaphthene	153	153.0697
Fluorene	166	165.0697
Dibenzothiophene	184	184.0339
Phenanthrene	178	178.0775
Anthracene	178	178.0775
4H-Cyclopenta[def]phenanthrene	189	189.0697
Fluoranthene	202	202.0774
Pyrene	202	202.0774
Cyclopenta[cd]pyrene	226	226.0775
Benz[a]anthracene	228	228.0932
Chrysene	228	228.0932
Triphenylene	228	228.0932
Benzo[b + j]fluoranthene	252	252.0934
Benzo[k]fluoranthene	252	252.0934
Benzo[a]fluoranthene	252	252.0934
Benzo[a]pyrene	252	252.0934
Benzo[e]pyrene	252	252.0934
Perylene	252	252.0934
Indeno[1,2,3-cd]pyrene	276	276.0935
Dibenzo[a,h]anthracene	278	278.1092
Benzo[ghi]perylene	276	276.0935
Coronene	300	300.0936
Naphthalene-D8	136	136.1122
Acenaphthylene-D8	160	160.1121
Acenaphthene-D10	162	162.1263
Fluorene-D10	174	174.1262
Phenanthrene-D10	188	188.1402
Anthracene-D10	188	188.1402
Fluoranthene-D10	212	212.1401
Pyrene-D10	212	212.1401
Benz[a]anthracene-D12	240	240.1684
Chrysene-D12	240	240.1684
Benzo[b]fluoranthene-D12	264	264.1685
Benzo[k]fluoranthene-D12	264	264.1685
Benzo[a]pyrene-D12	264	264.1685
Indeno[1,2,3-cd]pyrene-D12	288	288.1688
Dibenzo[a,h]anthracene-D14	292	292.1970
Benzo[ghi]perylene-D12	288	288.1688

### Analysis by GC-Orbitrap-MS

Sample extracts were injected (1 μL) into a GC Q-Exactive Orbitrap MS (GC-Orbitrap-MS) (Thermo Fischer Scientific, Whaltham, USA) equipped with the same column and GC program as the GC–MS. The resolution was set to 60,000 (FWHM; instrument setting at 200 u). The mass spectrometer was set to monitor the mass range between 50 and 650 u in EI + (70 eV) mode. Automated gain control (AGC target) was set to 1 × 10^6^, and maximum inject time was set to “auto”. The exact ion masses of the PAHs are given in Table 1. Quantification was performed with the same methodology as GC–MS (the “Analysis by conventional liquid extraction and GC–MS” section).

## Results and discussion

### Analytical quality control

The linearity of a five-point calibration curve for the SRM standard was tested between 0.5 and 500 pg (per 1 μL injected) using the ratio of the chromatographic peak area of the compounds to the area of the respective deuterated compounds (signal ratios), and the corresponding amount ratios. The expanded analytical uncertainties (EU%) of the standards were given by the suppliers, and this was combined with the standard errors of the consecutive dilution steps of the microbalance to obtain the different concentrations. This provided the expected uncertainties of all constituents in the standard mixtures (Table S1). The overall EU% of the calibration standards was highest for the lowest, 0.5 pg/μL, concentration (ranging from 5.4 to 7.5%), while the lowest EU% was calculated for the highest, 500 pg/μL, concentration (4.7 to 7.0%). These EU% were acceptably low and the small increase of EU% along dilution steps showed that the preparation of the standard mixtures of the calibration curve did not have a substantial contribution to the overall expected uncertainty.

The SRM2260a standard mixtures for the calibration curve were injected into the GC-Orbitrap-MS in two sessions that were separated by one month. The expected value is the average value of the two analyses (x), while the uncertainty (u) was calculated as u = (a1 – a2)/√12, were a1 and a2 are the (area/area-D) responses in the first and second analyses. The relative uncertainty (u%) was calculated as u% = (x/u) × 100. The expanded uncertainty (EU%) was two times the average u% of the five calibration points. One-to-one comparison of the signal ratios of the five concentration points in the two injection sessions showed average EU% that were lower than 14.9% except for benzo[a]fluoranthene (48.4%), biphenyl (23%), coronene (40.9%), and dibenzothiophene (41.6%) (Table S2). These results showed that the analysis of the standards by GC-Orbitrap-MS is accurate and precise for most PAHs within an acceptable uncertainty, except for the aforementioned four compounds.

The regression coefficients (*R*^2^) of the linear calibration curves for all tested compounds were higher than 0.999 (Fig. S2 shows the curve of benzo[a]pyrene), although the lowest concentration of the standards (0.5 pg/μL) could not be detected for biphenyl and coronene. These two PAHs, together with dibenzothiophene and benzo[a]fluoranthene, also showed higher analytical uncertainties, which could be due to the absence of their deuterated compounds in the surrogate standard. However, other compounds such as benzo[e]pyrene did not have an individual deuterated compound equivalent in the standard mixture and did not present this problem. Other properties such as volatility may also play a role, since biphenyl and coronene are the most and least volatile PAHs tested in the present study, respectively. The analytical error of the calibration curve of the other PAHs showed EU% of less than 1.7%, which is more than acceptable for analytical quality standards. Transformation of the calibration curve to a power regression curve was also tested, since this curve often results in smaller errors in the lower concentration range (Fig. S2). *R*^2^ of the power regression curves for the tested compounds were always higher than 0.999, showing that the good coefficients of the calibration curves were maintained. The EU% of the power regression calibration curves were also lower than 1.7%. Both types of regression curves proved that they can be used for the calibration and quantification of most PAH compounds with an acceptable uncertainty (EU < 15%).

So, the GC-Orbitrap-MS is sensitive enough to detect and quantify the PAHs in full scan mode at the lowest concentration of 0.5 pg/μL injected into the instrument with high precision and accuracy. This concentration is about ten times lower than in the conventional single quadrupole GC–MS analysis, although it is still ten times higher than PAH analysis by GC-APLI-TOF–MS (Stader et al., 2013). Nevertheless, the quantification limit of the GC-Orbitrap-MS method applies to concentrations of whole sample extracts, which are often diluted in 25 μL end-volume in vials. This implies that environmental samples with low PAH concentrations can be analyzed as whole filters, or that small fractions (and volumes) of the whole filter with high PAH loads can be analyzed. This is very relevant in exposure and risk-assessment studies that determine the impact of PAHs on human health, especially in environments with ambient air concentrations that exceed air quality standards. For example, the most toxic PAH, benzo[a]pyrene, has an annual target value of 1000 pg/m^3^ in outdoor ambient air PM in EU countries (2004/107/EC), although the World Health Organization considers 120 pg/m^3^ as the reference level for human exposure, corresponding to an excess lifetime cancer risk of 1/100,000 (WHO, 2017). In a high-volume PM sampler (24 h at 30 m^3^/h), this latter concentration results in more than 80,000 pg of benzo[a]pyrene in the whole filter sample matrix, evidencing that a small sub-cubic meter fraction would be enough to be analyzed above the quantification limit of the GC-Orbitrap-MS. Another general advantage of the analysis of small aliquots of PM samples is the reduction of interferences from the matrix and blank levels, thus maintaining low quantification limits. Furthermore, these smaller samples allow the reduction of purification and concentration steps in the analytical procedure, saving time, and consumables.

### Aliquot analysis of high-volume PM samples on PAHs by GC-Orbitrap-MS

**Fig. 2:**
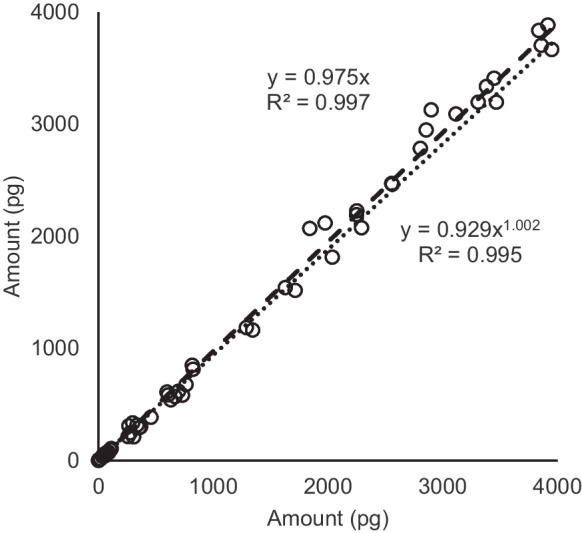
Amount (pg in extract) of all analyzed PAH compounds in three duplicate PM2.5 samples for repeatability test in GC-Orbitrap-MS

In order to illustrate the possibilities of analyzing small volume samples, sections of high volume PM2.5 quartz filter samples were taken with a punch cutter (Ø = 3.4 mm; equivalent sample volumes of 0.37 m^3^; Fig. 1). These aliquots were extracted in the vial containing the surrogate standard in cyclo-hexane and directly analyzed on the GC-Orbitrap-MS without any further purification and concentration steps in the procedure. For this test, fourteen PM samples and two field blanks were collected in a semi-rural area (Manlleu). This site often showed benzo[a]pyrene concentrations above 1 ng/m^3^ in winter (Gencat, 2022). The elevated PAH concentrations in this area are related to biomass burning emissions in combination with stagnant atmospheric conditions (Jaén et al., 2021). Three of the samples were analyzed in duplicate in order to test the repeatability of the analytical procedure. All PAHs with molecular masses higher than 202 m/z (from fluoranthene to coronene) were detected in the punch samples, but none of them were detected in the field blank filters. Coronene, benzo[a]fluoranthene, and dibenzo[a,h]anthracene were only detected in the sample with highest PAH load. The EU% of the repeatability test ranged from 22.1% (coronene) to 3.4% (Fig. 2; Table 2), showing that the analytical procedure was highly repeatable despite the use of very small sample fractions. There were no substantial differences between the EU% in the sample with lower PAH load (ΣPAH = 5000 pg/sample) compared to the sample with highest PAH load (ΣPAH = 35,000 pg/sample).

**Table 2:** Relative uncertainties of duplicate punch sample (0.37 m^3^) by GC-Orbitrap-MS analysis, based on a one-to-one comparison of PAH compound concentrations

Compound	**EU%**
Dibenzothiophene	4.9
Phenanthrene	5.0
Anthracene	9.2
4H-Cyclopenta[def]phenanthrene	9.3
Fluoranthene	6.3
Pyrene	3.8
Cyclopenta[cd]pyrene	5.8
Benz[a]anthracene	5.3
Chrysene	3.9
Triphenylene	6.7
Benzo[b]fluoranthene	4.7
Benzo[j + k]fluoranthene	6.9
Benzo[a]fluoranthene	10.9
Benzo[e]pyrene	4.4
Benzo[a]pyrene	4.9
Perylene	2.9
Indeno[1,2,3-cd]pyrene	5.9
Dibenzo[a,h]anthracene	6.6
Benzo[ghi]perylene	3.4
Coronene	22.1

**Fig. 3:**
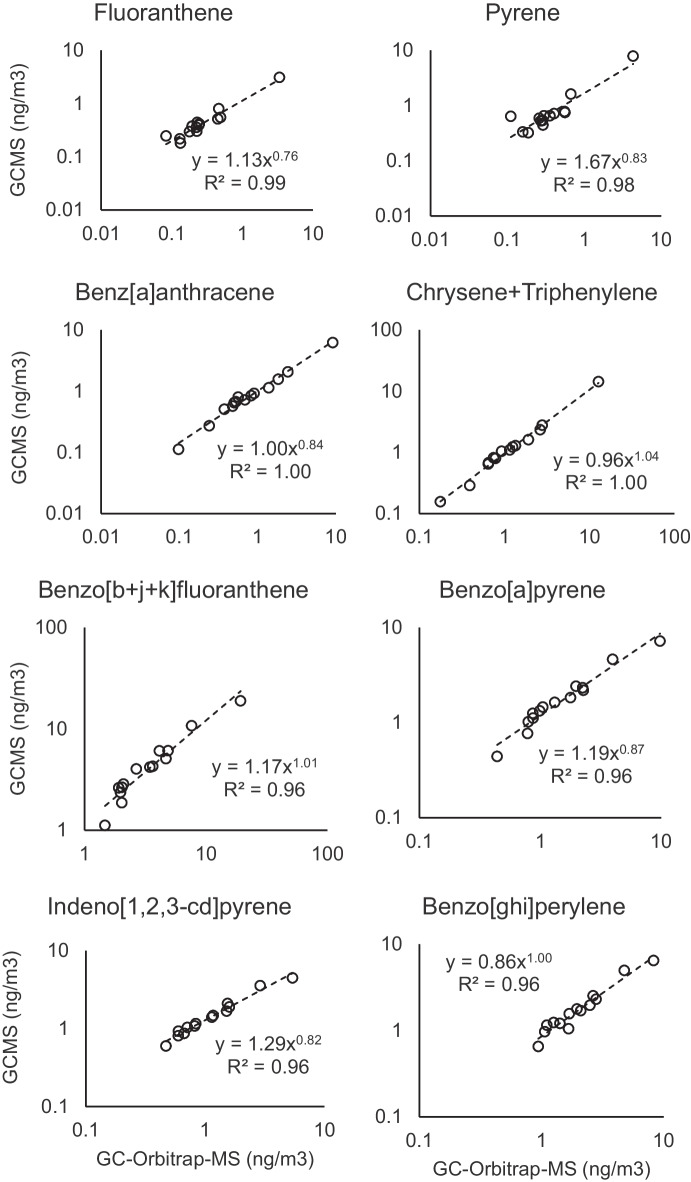
PAH concentrations (ng/m^3^ in log scale) in 1/4 filter sample extraction and GC–MS analysis (180 m^3^) versus punch sample GC-Orbitrap-MS analysis (0.37 m^3^)

The measured PAH concentrations in the PM2.5 punch samples by GC-Orbitrap-MS in full scan mode were compared with the results obtained by analysis of 1/4 of the filter sample (equivalent sample volume of 180 m^3^) by single quadrupole GC–MS analysis in SIM mode. The concentrations of the three isomers benzo[b + j + k]fluoranthene, as well as chrysene and triphenylene, were summed since they were not well resolved chromatographically in the GC–MS analysis. Figure 3 shows the PAH concentrations obtained by these analytical methods and the regression correlations of the quantified compounds. Both analytical methods showed EU% between 5 ± 5% and 15 ± 5% for the quantified PAHs, except for fluoranthene (25 ± 14%) and pyrene (37 ± 16%). Higher errors could be expected for these compounds since they are more volatile than the other PAHs and more sensitive to losses during the two analytical procedures. PAHs that are more, or completely, particle-bound (benz[a]anthracene to benzo[ghi]perylene), showed acceptable uncertainties and good reproducibility between the methods. For example, benzo[a]pyrene, with sample concentrations between 0.4 and 10 ng/m^3^, showed an EU% between the two methods of 10 ± 8%, indicating that the GC-Orbitrap-MS analysis of the small filter sample fractions provided reproducible results with respect to the GC–MS analysis. The benzo[a]pyrene concentrations in the samples were in the range of the concentrations analyzed by regional authorities in other samples from the same period in the semi-rural site of Manlleu (Fig. S3).

### PAH analysis in micro-Aethalometer AE51 filter strips

**Fig. 4:**
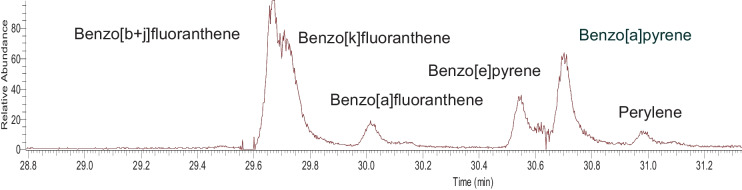
GC-Orbitrap-MS fragmentogram of the exact mass for benzofluorantenes, benzopyrenes, and perylene (m/z 252.0934) in a punch sample (0.144 m^3^) of the Aetahlometer AE51 filter strip collected during 24 h in a village in the Pyrenees

The high sensitivity and accuracy of the GC-Orbitrap-MS method for PAH analysis opens the possibility to determine these compounds on small sample fractions, such as those on the filter strips of the Aethalometer AE51. Filter strips from these portable real-time eBC analyzers were used successfully in indoor PAH analysis in former studies using GC-APLI-TOF–MS (Yan et al., 2018). Here, the filter strips were analyzed for outdoor particle-bound PAHs (benz[a]anthracene to benzo[ghi]perylene) in rural and urban areas after 24-h eBC monitoring (total sample volume = 0.144 m^3^) and after 5-h personal exposure monitoring (total sample volume = 0.030 m^3^) of firefighters during a prescribed forest burn. No further purification and concentration steps were undertaken in the analytical procedure, other than ultrasonic bath extraction of the punch sample in 25 μL of surrogate standard in cyclohexane. Figure 4 shows a chromatogram of a selection of PAHs in a low-volume filter strip sample.

**Fig. 5:**
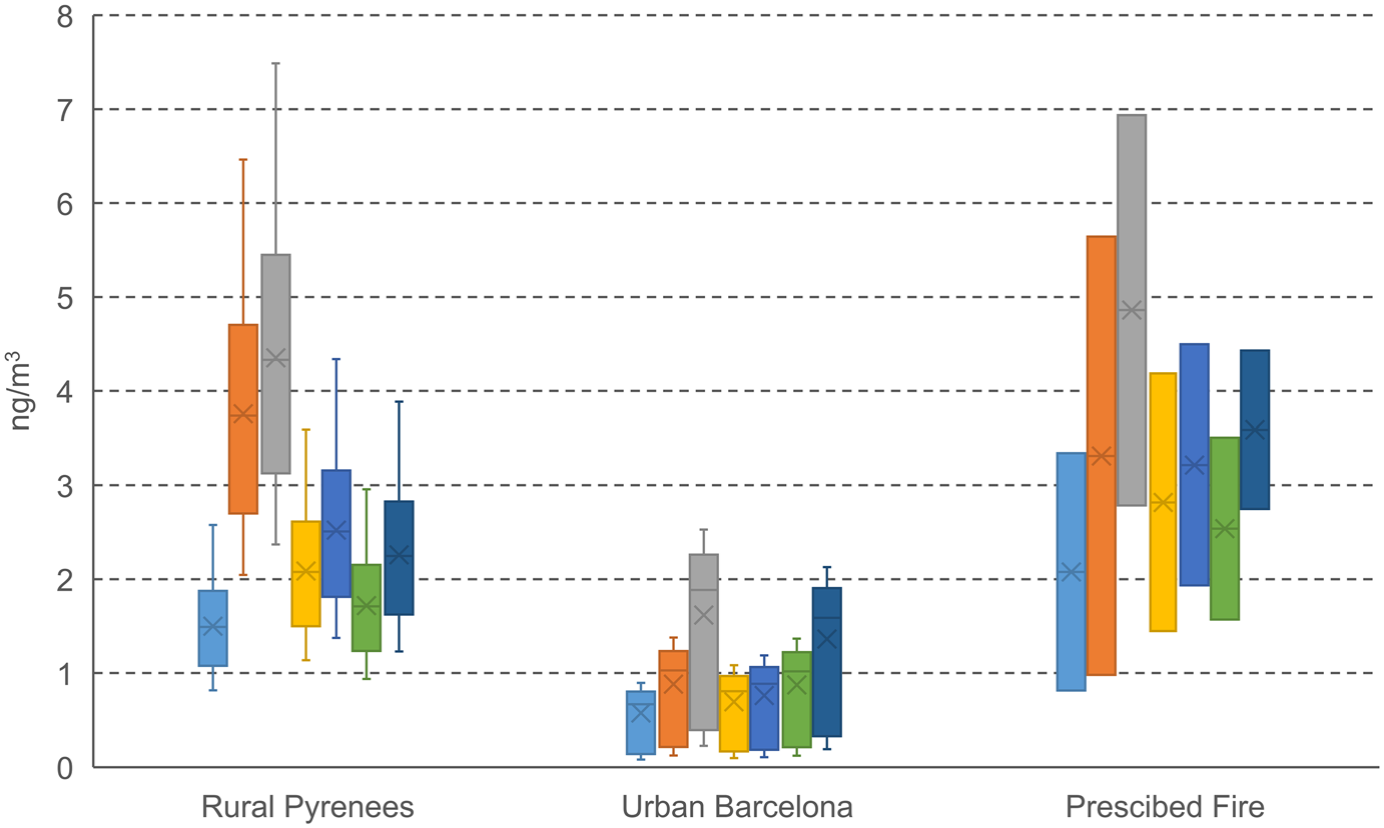
Concentration (ng/m^3^) of PAHs (from left to right: benz[a]anthrence, chrysene + triphenylene, benzo[b + j + k]fluoranthene, benzo[e]pyrene, benzo[a]pyrene, indeno[1,2,3-cd]pyrene, benzo[ghi]perylene) analyzed in Aethalometer AE51 filter strips (0.144 m^3^) collected in a village in the Pyrenees (*n* = 11) and an urban traffic site in Barcelona (*n* = 19) in wintertime, and a prescribed forest fire in Avinyó in fall (*n* = 2)

**Fig. 6:**
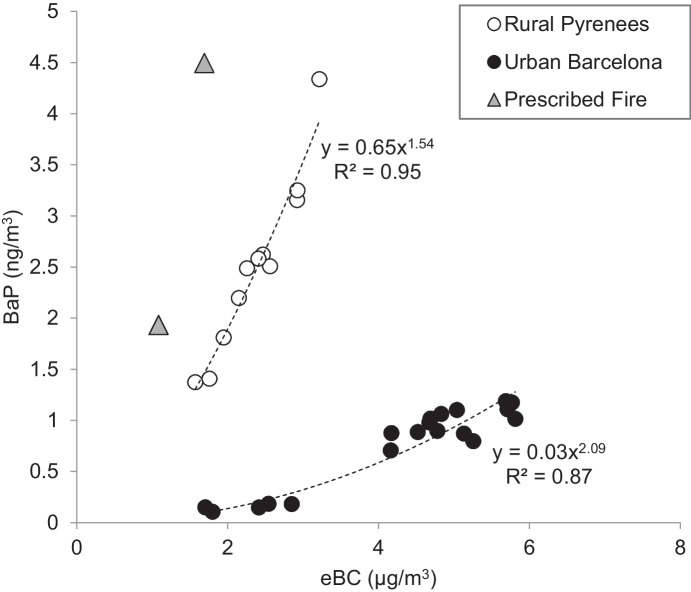
Benzo[a]pyrene (BaP) concentrations analyzed in filter strips versus equivalent black carbon (eBC) average concentrations measured with the Aethalometer AE51 in a village in the Pyrenees, an urban site in Barcelona, and a prescribed forest fire site in Avinyó

All PAH compounds except dibenzo[a,h]anthracene were above the limit of 0.5 pg/μL in all samples. In the rural samples from a village in the Pyrenees, the concentrations of benzo[a]pyrene ranged between 1.4 and 4.3 ng/m^3^ (mean = 2.5 ng/m^3^), while they were between 0.2 and 1.2 ng/m^3^ (mean = 0.8 ng/m^3^) in the urban samples from Barcelona. This generally higher impact of PAHs in rural areas in winter is due to biomass burning from domestic heating and stagnant weather conditions. The composition in the rural samples was dominated by benzofluoranthenes, chrysene + triphenylene, and benzo[a]pyrene, while in the urban samples they were dominated by benzofluoranthenes and benzo[ghi]perylene (Fig. 5). PAH concentrations and compositions are in agreement with previous studies in these sites (Jaén et al., 2021; van Drooge & Grimalt, 2015). The quantification of PAHs in the AE51 filter strips allowed the comparison with the measured real-time eBC concentrations, averaged over the whole sampling time. We observed good correlations between eBC and PAH concentrations, although the rural and urban sites showed independent slopes (Fig. 6). These differences can be linked to the nature of the equivalent black carbon and the light absorption of the AE51. Biomass burning, especially at low temperature, generates char and more PAHs, but less soot (black) carbon, while vehicle engines generate more soot carbon and less PAHs at high-temperature combustion (Fine et al., 2004; Sandradewi et al., 2008; Schauer et al., 2008). The light adsorption of soot (black) carbon is optimal at 880 nm, while the optimal wavelength for biomass smoke is around 375 nm [24]. The Aethalometer AE51 measured the rate of change in absorption of transmitted light at 880 nm, so it probably underestimated the char contributions from the biomass smoke. Nevertheless, the instrument collected PM2.5 on the filter strip, and these particles were analyzed for PAHs.

During the prescribed burn, two Aethalometer AE51, with eBC measurement uncertainties of less than 5% between them, were used as personal exposure monitoring instruments by firefighters. Small young trees (*Pinus halepensis*) and plants from a Mediterranean pine tree forest were burnt. One Aethalometer was worn by a firefighter that was directly involved in the prescribed burn (fire front or holding), while the other instrument was worn by a firefighter that was less exposed to smoke in the periphery of the prescribed fire, resulting in peak eBC concentrations of 635 and 135 μg/m^3^, respectively, and 5-h mean eBC concentrations of 1.7 and 1.1 μg/m^3^. PAHs were detectable in the filter strips of both samples, with relative compositions and sample concentrations similar to the one observed in the rural area exposed to biomass burning smoke (Fig. 5). Higher PAH concentrations were measured for the firefighter in the front line of the prescribed burn, with a benzo[a]pyrene concentration of 4.5 ng/m^3^ compared to 1.9 ng/m^3^ for the firefighter in the periphery. These levels indicate that firefighters are exposed to significant eBC and PAH concentrations during prescribed burns, which is probably also the case during other activities around wildfires. Firefighters do not wear protection masks, or respiratory equipment, which makes these exposure concentrations even more relevant. Hence, presence of PAHs in wildfire smoke is one of the reasons that occupational exposure as a firefighter was classified as “carcinogenic to humans” (group 1) (Demers et al., 2022). Despite the limited amount of samples, the measured eBC and PAH concentrations show important variation of exposure concentrations during the occupational tasks, which was also observed in other studies (Navarro et al., 2017), and it is obvious that more samples are required to get a better picture on the occupational PAH exposure of firefighters. In former studies, attempts were made to determine the personal exposure concentrations of PAHs for firefighters, but this was only partially achieved mainly due to high analytical method quantification limits for individual PAH compounds (Cherry et al., 2021; Navarro et al., 2017). In the present study, using the GC-Orbitrap-MS method and 5-h Aethalometer sample volume, the quantification limit was low enough to quantify the PAHs in the filter strips. Based on the eBC peak concentrations at 5-min monitoring resolution, the direct exposure to high concentrations of smoke particles and PAHs was probably even less than 1.5 h. Despite this short exposure period, the measured PAH concentrations were in the range of those observed in other studies of personal exposure in firefighters. In fact, the sum of five- and six-ring PAHs were 10 and 24 ng/m^3^ in our samples, which is comparable to those observed in PM in other prescribed fires (25 ng/m^3^) (Navarro et al., 2017).

The PAH analysis in AE51 filter strips by GC-Orbitrap-MS can therefore be used to increase the knowledge on the possible dominating combustion sources in outdoor air and personal exposure monitoring. On the other hand, the real-time eBC concentrations are indicative of the levels of benzo[a]pyrene and other PAHs in the ambient air PM. Using the present approach, these concentrations can be estimated at temporal resolutions of 5 min, corresponding to the resolution of the eBC measurements by the Aethalometer AE51. Based on the correlations between eBC and PAH observed in the present study in the rural and urban sites (Fig. 6), the average 24-h eBC level in the rural area exposed to biomass burning, and wildfire smoke exposure, should be higher than 0.3 μg/m^3^ in order to quantify individual PAHs (> 0.5 pg/μL injected). On the other hand, the eBC level should be higher than 1.8 μg/m^3^ in urban sites (exposure to combustion vehicle engine exhaust). In many European cities, the mean eBC concentrations are above this level (Querol et al., 2013; Reche et al., 2011), showing that the Aethalometer AE51 analysis and consecutive PAH analysis by GC-Orbitrap-MS can be a very useful tool to determine PAH levels in PM samples quickly, precisely, and accurately.

## Conclusions

The GC-Orbitrap-MS instrument provides significant improvements for the analysis of PAHs at trace levels in atmospheric particulate matter (PM). In the present study, this instrument has been tested successfully for the analysis of PAHs in standard reference material (SRM2260a) and ambient air particulate matter (PM2.5) in full scan mode. The results are comparable to those obtained by conventional GC–MS analysis. The analytical uncertainties for the particulate-bound PAHs are in the range of acceptable analytical quality standards, showing high precision and accuracy. The quantification limit of the GC-Orbitrap-MS method for full scan analysis of PAHs in PM samples was 0.5 pg/μL. These limits are ten times lower than those obtained by conventional single quadrupole GC–MS analysis. The low quantification limit of the GC-Orbitrap-MS allows the analysis of small sample fractions and/or samples with short sampling times, since outdoor ambient air PM concentrations for individual PAHs range between a few pg/m^3^ and several ng/m^3^. The obtention of section samples (Ø = 3.4 mm) from a whole PM filter sample, consecutive extraction in surrogate standard solution, and direct analysis by GC-Orbitrap-MS are found to be efficient, accurate, and precise. The method provides faster acquisition of data on ambient air PAH concentrations than the conventional GC–MS method, saving time and consumables in the analysis. Moreover, only a very small fraction of the whole filter sample is used for analysis, which offers the possibility of further analyses in these filters, such as toxicity studies. PAHs were detected and quantified successfully in filter strips of portable Aethalometers for real-time equivalent black carbon monitoring, adding synchronized data on these compounds to the real-time ambient air and personal exposure monitoring measurements. The GC-Orbitrap MS method is currently being tested for the detection and quantification of other toxic PAH derivatives, such as N-PAH and O-PAH, as well as molecular tracer compounds for emission source identification.

## Supplementary Information

Below is the link to the electronic supplementary material.

Supplementary file1 (DOCX 860 KB)

## Data Availability

The datasets generated during and/or analyzed during the current study are available from the corresponding author on reasonable request.
