# Supplementary material for: Determination of subpicogram levels of airborne polycyclic aromatic hydrocarbons for personal exposure monitoring assessment
Source: Environ Monit Assess. 2023 Feb 7;195(3):368. doi: 10.1007/s10661-023-10953-z (PMC9905180; doi:10.1007/s10661-023-10953-z)
Supplement: Supplementary file 1 — Supplementary file1 (DOCX 860 KB) [file 10661_2023_10953_MOESM1_ESM.docx]

**SUPPLEMENTARY INFORMATION**

**Determination of subpicogram levels of airborne polycyclic aromatic hydrocarbons for personal exposure monitoring assessment**

Barend L. van Drooge, Raimon M. Prats, Clara Jaén Flo, Joan O. Grimalt

Institute of Environmental Assessment and Water Research, Spanish National Research Council (IDÆA-CSIC). c/Jordi Girona 18-26, 08034. Barcelona, Spain.

Table S1. Relative uncertainties of the deuterated compounds and PAH compounds in the SRM2260a including the uncertainties (u%) of the compounds and analytical uncertainties from the micro-balance (1%) in consecutive dilution steps. The expanded error (EU%) is two times the average overall uncertainty of the calibration standard, expressed as EU% = 2 x √(u_deut_^2^ + u_SRM_^2^). The deuterated compounds that were used for the calibration curves are placed in front of the corresponding PAH compound (for example, naphthalene-D8 was used for biphenyl).

| Calibration curve | u% | Compound | 0.5 pg | 2.5 pg | 5 pg | 50 pg | 500 pg | **EU%** |
| --- | --- | --- | --- | --- | --- | --- | --- | --- |
| Naphthalene-D8 | 1.5 | Biphenyl | 2.8 | 2.8 | 2.7 | 2.6 | 2.5 | 6.2 |
| Naphthalene-D8 | 1.5 | Naphthalene | 3.0 | 2.9 | 2.9 | 2.8 | 2.7 | 6.4 |
| Acenaphthylene-D8 | 1.2 | Acenaphthylene | 2.9 | 2.9 | 2.8 | 2.7 | 2.6 | 6.1 |
| Acenaphthene-D10 | 1.1 | Acenaphthene | 2.7 | 2.6 | 2.6 | 2.5 | 2.4 | 5.6 |
| Fluorene-D10 | 1.2 | Fluorene | 2.8 | 2.7 | 2.7 | 2.6 | 2.4 | 5.8 |
| Phenanthrene-D10 | 1.2 | Dibenzothiophene | 3.4 | 3.3 | 3.3 | 3.2 | 3.1 | 6.9 |
| Phenanthrene-D10 | 1.2 | Phenanthrene | 2.5 | 2.4 | 2.4 | 2.3 | 2.2 | 5.3 |
| Anthracene-D10 | 1.2 | Anthracene | 2.5 | 2.5 | 2.4 | 2.3 | 2.2 | 5.3 |
| Phenanthrene-D10 | 1.2 | 4H-Cyclopenta[def]phenanthrene | 3.5 | 3.5 | 3.4 | 3.4 | 3.3 | 7.2 |
| Phenanthrene-D10 | 1.2 | Fluoranthene | 2.5 | 2.4 | 2.4 | 2.3 | 2.1 | 5.3 |
| Pyrene-D10 | 1.3 | Pyrene | 2.6 | 2.5 | 2.4 | 2.3 | 2.2 | 5.5 |
| Benz[a]anthracene-D12 | 1.6 | Cyclopenta[cd]pyrene | 2.7 | 2.6 | 2.6 | 2.5 | 2.4 | 6.0 |
| Benz[a]anthracene-D12 | 1.6 | Benz[a]anthracene | 2.7 | 2.7 | 2.6 | 2.5 | 2.4 | 6.1 |
| Chrysene-D12 | 1.3 | Chrysene | 2.8 | 2.7 | 2.7 | 2.6 | 2.5 | 5.9 |
| Chrysene-D12 | 1.3 | Triphenylene | 3.2 | 3.1 | 3.1 | 3.0 | 2.9 | 6.6 |
| Benzo[b]fluoranthene-D12 | 1.6 | Benzo[b]fluoranthene | 2.7 | 2.7 | 2.6 | 2.5 | 2.4 | 6.1 |
| Benzo[k]fluoranthene-D12 | 1.6 | Benzo[j+k]fluoranthene | 2.7 | 2.6 | 2.6 | 2.5 | 2.4 | 6.0 |
| Benzo[k]fluoranthene-D12 | 1.6 | Benzo[a]fluoranthene | 2.4 | 2.3 | 2.2 | 2.1 | 2.0 | 5.4 |
| Benzo[a]pyrene-D12 | 1.6 | Benzo[e]pyrene | 2.7 | 2.7 | 2.6 | 2.5 | 2.4 | 6.1 |
| Benzo[a]pyrene-D12 | 1.6 | Benzo[a]pyrene | 3.0 | 2.9 | 2.9 | 2.8 | 2.7 | 6.5 |
| Benzo[a]pyrene-D12 | 1.6 | Perylene | 2.7 | 2.6 | 2.6 | 2.5 | 2.4 | 6.1 |
| Indeno[1,2,3-cd]pyrene-D12 | 1.6 | Indeno[1,2,3-cd]pyrene | 2.7 | 2.6 | 2.6 | 2.5 | 2.3 | 6.0 |
| Dibenzo[a,h]anthracene-D14 | 1.1 | Dibenzo[a,h]anthracene | 2.5 | 2.4 | 2.3 | 2.2 | 2.1 | 5.1 |
| Benzo[ghi]perylene-D12 | 1.2 | Benzo[ghi]perylene | 2.5 | 2.4 | 2.4 | 2.3 | 2.2 | 5.3 |
| Benzo[ghi]perylene-D12 | 1.2 | Coronene | 2.5 | 2.5 | 2.4 | 2.3 | 2.2 | 5.3 |

Table S2. Relative uncertainty of the calibration curve was calculated from two analyses that were separated by one month, based on a one-to-one comparison of the five standard concentrations that were injected in GC-Orbitrap-MS. nd = not detected.

| Compound | 0.5 pg | 2.5 pg | 5 pg | 50 pg | 500 pg | **EU%** |
| --- | --- | --- | --- | --- | --- | --- |
| Biphenyl | nd | 21.5 | 18.8 | 5.3 | 0.4 | 23.0 |
| Naphthalene | 2.2 | 0.3 | 0.0 | 1.3 | 0.6 | 1.7 |
| Acenaphthylene | 3.9 | 3.6 | 1.5 | 0.8 | 0.2 | 4.0 |
| Acenaphthene | 0.5 | 1.8 | 0.4 | 0.2 | 0.3 | 1.3 |
| Fluorene | 2.5 | 1.8 | 0.0 | 1.2 | 0.1 | 2.2 |
| Dibenzothiophene | 53.5 | 20.5 | 20.1 | 7.0 | 2.7 | 41.6 |
| Phenanthrene | 3.9 | 0.6 | 0.4 | 0.0 | 0.3 | 2.1 |
| Anthracene | 1.5 | 6.5 | 7.3 | 6.5 | 1.9 | 9.5 |
| 4H-Cyclopenta[def]phenanthrene | 0.2 | 2.2 | 3.3 | 4.0 | 3.0 | 5.1 |
| Fluoranthene | 6.5 | 1.4 | 1.7 | 0.2 | 0.5 | 4.2 |
| Pyrene | 6.3 | 2.6 | 0.3 | 1.2 | 0.2 | 4.3 |
| Cyclopenta[cd]pyrene | 12.7 | 7.4 | 5.3 | 2.8 | 1.9 | 12.0 |
| Benz[a]anthracene | 2.3 | 2.4 | 1.8 | 0.8 | 0.3 | 3.0 |
| Chrysene | 1.6 | 1.3 | 1.4 | 0.6 | 0.7 | 2.3 |
| Triphenylene | 0.8 | 0.3 | 0.9 | 1.9 | 1.6 | 2.2 |
| Benzo[b]fluoranthene | 7.7 | 4.1 | 1.4 | 1.7 | 1.7 | 6.7 |
| Benzo[j+k]fluoranthene | 5.5 | 2.4 | 3.0 | 1.2 | 0.3 | 5.0 |
| Benzo[a]fluoranthene | 28.9 | 40.6 | 31.7 | 12.2 | 7.5 | 48.4 |
| Benzo[e]pyrene | 5.2 | 3.9 | 5.4 | 2.9 | 2.6 | 8.0 |
| Benzo[a]pyrene | 3.2 | 0.0 | 2.9 | 2.3 | 2.9 | 4.5 |
| Perylene | 9.9 | 3.4 | 1.2 | 2.7 | 0.9 | 7.3 |
| Indeno[1,2,3-cd]pyrene | 28.9 | 3.1 | 1.4 | 2.6 | 1.2 | 14.9 |
| Dibenzo[a,h]anthracene | 12.1 | 4.4 | 0.9 | 1.6 | 1.2 | 8.1 |
| Benzo[ghi]perylene | 21.6 | 6.0 | 1.7 | 2.4 | 0.4 | 12.8 |
| Coronene | nd | nd | 28.9 | 20.5 | 11.9 | 40.9 |


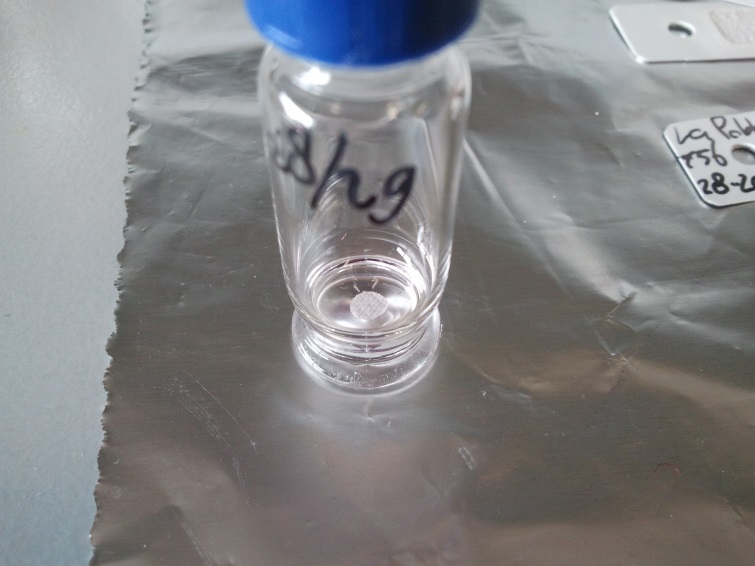

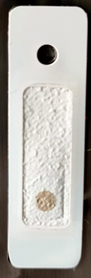


Figure S1. Aethalometer AE51 filter strip and sample punch fraction (Ø = 3.4 mm; 0.144 m^3^) in analytical vial.

|  |
| --- |

Figure S2. Calibration curve of benzo[a]pyrene of the two analyses by GC-Orbitrap-MS, where the y-axis represents the area/area-D and x-axis the amount/amount-D.

|  |
| --- |

Figure S3. Benzo[a]pyrene concentrations in punch samples from PM2.5 filters collected in Manlleu (semi-rural site in Spain). Triangles are GC-Orbitrap-MS measurements and black dots are measurements done by the regional authorities (Generalitat de Catalunya).
